# Increased incidence of malignancy in patients with primary hyperparathyroidism

**DOI:** 10.3906/sag-2012-18

**Published:** 2021-08-30

**Authors:** Melia KARAKÖSE, Muhammet KOCABAŞ, Mustafa CAN, Hatice ÇALIŞKAN BURGUCU, İlker ÇORDAN, Mustafa KULAKSIZOĞLU, Feridun KARAKURT

**Affiliations:** 1 Department of Endocrinology and Metabolism, Faculty of Medicine, Necmettin Erbakan University, Konya Turkey; 2 Department of Endocrinology and Metabolism, Konya City Hospital, Konya Turkey

**Keywords:** Primary hyperparathyroidism, cancer, thyroid cancer

## Abstract

**Background/aim:**

Primary hyperparathyroidism (PHPT) is a disease that is diagnosed more frequently and generally in the asymptomatic period, with widely available biochemical tests. Evidence suggesting an association between PHPT and malignancy risk is increasing. Clarification of this association will be useful in PHPT for malignancy screening and management of patients with PHPT. In this study, we aimed to investigate the frequency of cancer in PHPT patients.

**Materials and methods:**

A total of 775 PHPT patients were included in the retrospective study. Demographic, clinical and laboratory data of the patients were evaluated retrospectively.

**Results:**

Malignancy was detected in 128 (16.50%) of 775 PHPT patients (female/male: 625/150). The mean age at diagnosis of PHPT was 57.99 ± 10.86 years, and the mean age at diagnosis of malignancy was 57.46 ± 11.17 years. Of the 128 patients with malignancy, 53 (41.40%) were diagnosed in the same year as PHPT. In terms of malignancy types, 51 (6.50%) of 775 PHPT patients had thyroid cancer. Thyroid cancer was followed by breast cancer (2.30%) and stomach cancer (1%) in order of frequency.

**Conclusion:**

We think that PHPT patients should be examined more carefully in terms of cancer risk, especially thyroid cancer. More comprehensive studies are needed to clarify the relationship between PHPT and cancer.

## 1. Introduction

Primary hyperparathyroidism (PHPT) is a disease characterized by hypercalcaemia and elevated or inappropriately normal serum level of parathyroid hormone (PTH) due to parathyroid adenoma, hyperplasia or rarely cancer. As biochemical tests are widely available, PHPT has become a disease that is diagnosed more frequently and generally in the asymptomatic period today. PHPT is a relatively common endocrine disease with an incidence of up to 1 in 1000 patients. PHPT can be seen at all ages, but it is most common in the sixth decade of life; women are affected three times more often than men [1–2]. Half of all patients with PHPT are postmenopausal women [3]. Its treatment requires surgical resection of the related parathyroid gland or glands.

Increasing incidence of PHPT and good prognosis have raised concerns about many different types of cancer that occur during the follow-up of these patients. In addition, PHPT can occur in patients who are followed for any cancer. There are studies reporting that many types of cancer such as breast, kidney, colon, skin, pancreas, lung, hematopoietic (especially multiple myeloma), pituitary, thymus, adrenal and thyroid are more common in patients with PHPT than in the general population [4–7]. In terms of the frequency of individual cancer types in PHPT patients, it is seen that there are more studies showing that the frequency of thyroid cancer has increased in those patients [8–10].

Evidence suggesting an association between PHPT and malignancy risk is increasing. The clarification of this association will provide useful information for malignancy screening in patients with PHPT and long-term management of those patients. In this study, we aimed to investigate the frequency of cancer in PHPT patients.

## 2. Materials and methods

A total of 775 patients who were followed-up with the diagnosis of PHPT between 2006 and 2019 were included in this retrospective study. Necmettin Erbakan University Meram Medical School Ethics Committee approved the study. PHPT complications such as osteoporosis and nephrolithiasis, the demographic, clinical, laboratory, radiological and pathological data of the patients were documented from the hospital’s electronic registry. The presence of an accompanying malignancy, ages at the time of diagnosis of PHPT and malignancy were questioned.

### 2.1. Statistical analysis

Statistical analyses were performed using the SPSS 22.0 (Statistical Package for Social Sciences, IBM Corp., Armonk, NY, USA) program. Continuous variables were given as mean ± standard deviation if the distribution was normal and as median (minimum-maximum) if the distribution was not normal. In the comparison of independent group differences, the significance test of the difference between the two means (independent samples t test) was used when the parametric test assumptions are provided. The Mann–Whitney U test was used to compare the independent group differences when the parametric test assumptions were not provided. For differences, p < 0.05 value was considered statistically significant.

## 3. Results

The data of 775 patients with PHPT were analyzed in this study. 625 (80.6%) of the 775 patients were female, and 150 (19.4%) patients were male. 128 (16.5%) of 775 PHPT patients were found to have a diagnosis of malignancy. The mean age of the study population was 57.99 ± 10.86 years at the time of the diagnosis of the PHPT and 57.46 ± 11.17 years at the time of the diagnosis of the accompanying malignancy. The median follow-up was 5 (1–13) years for PHPT and 5 (1–14) years for accompanying malignancy.

Parathyroid adenoma could be localized in 74.5% (577/775) of patients and based on ultrasonography, the **parathyroid adenomas** were located on the right inferior in 46.1% of patients, left inferior in 41.0% of patients, right superior in 5.0% of patients, left superior in 4.8% of patients and medistinal in 1.5% of patients. Laboratory evaluation showed that the median level of serum calcium (Ca) was 11.20 (10.00–15.80) mg/dL, albumin-corrected Ca was 11.13 (9.36–16.80) mg/dL, PTH was 173 (68 –1604) pg/mL, albumin was 4.3 (2.5–5.5) g/dL, phosphorus (P) was 2.7 (0.9–6.2) mg/dL. The median 24-h urinary Ca excretion was 310 (100–1568) mg/day. In females, serum Ca was 11.00 (10.00–15.80) mg/dL, albumin-corrected Ca was 11.05 (10.00–16.80) mg/dL, P was 2.72 (1.10V 5.60) mg/dL, albumin was 4.3 (3.2–5.2) g/dL and 24-h urinary Ca excretion was 331 (100–1568) mg/day (reference range < 250 mg/day), while, in males, serum Ca was 11.61 (10.00–15.40) mg/dL, albumin-corrected Ca was 11.44 (10.00–15.77) mg/dL, P was 2.50 (0.90–6.20) mg/dL, albumin was 4.2 (2.5–5.5) g/dL and 24-hour urinary Ca excretion was 409 (101–956) mg/day (reference range < 300 mg/day). According to the dual-energy X-ray absorptiometry (DEXA) and urinary ultrasonography results, 32.9% of patients had **osteoporosis** and 17.1% had **nephrolithiasis** (Table 1). Frequency of nephrolithiasis was significantly higher in men than in women (31.6% in men and 13.4% in women, p < 0.001). There was no difference between men and women in terms of the frequency of osteoporosis (25.8% in men and 34.1% in women, p = 0.415).

**Table 1:** Demographic, clinical and biochemical data.

Age at diagnosis (PHPT), year, mean ± SD	57.99 ± 10.86
Age at diagnosis (malignancy), year, mean ± SD	57.46 ± 11.17
Follow-up time (PHPT), year, median (min-max)	5 (1–13)
Follow-up time (malignancy), year, median (min-max)	5 (1–14)
Sex, male, n (%)	150 (19.4)
Ca, mg / dL, median (min-max)	11.20 (10.00–15.80)
Albumin-corrected Ca (min-max)	11.13 (9.36–16.80)
P, mg / dL, median (min-max)	2.70 (0.90–6.20)
PTH, ng / L, median (min-max)	173 (68–1604)
Vitamin D, ug / L, median (min-max)	12 (3–52)
Albumin, g / dL, median (min-max)	4.3 (2.5–5.5)
ALP, U / L, median (min-max)	91 (31–934)
24-hour urine calcium, mg / day, median (min-max)	310 (100–1568)
Urinary calcium elevation (n: 488), n (%)	260 (46.7)
Nephrolithiasis (n: 384), n (%)	66 (17.1)
Osteoporosis (n: 213), n (%)	70 (32.9)
Frequency of malignancy, n (%)	128 (16.5)

A total of 128 (16.5%) of 775 PHPT patients were found to have a diagnosis of malignancy. The frequency of malignancy in PHPT patients was 29/150 (19.3%) in men and 99/625 (15.8%) in women, but this difference did not reach statistical significance (p = 0.327). Of the 128 patients with malignancy, 53 (41.4%) were diagnosed in the same year as PHPT, 33 (25.7%) were diagnosed before PHPT, and 42 (32.8%) after PHPT. In terms of malignancy types, 51 (6.5%) of 775 PHPT patients had thyroid cancer (48 papillary, 2 follicular, 1 medullary), and 27 of these 51 thyroid cancer patients had the diagnosis of thyroid cancer and PHPT simultaneously (Table 2, Figure).

**Table 2:** Cancer types seen in PHPT patients (n: 775).

	n	%		n	%
All types of cancer	128	16.5	Squamous cell skin cancer	3	0.4
Thyroid cancer	51	6.5	Colon cancer	3	0.4
Breast cancer	18	2.3	Renal cell cancer	3	0.4
Stomach cancer	8	1	Cervical cancer	2	0.3
Polisitemia vera	7	0.9	Adenocarcinoma of unknown primary origin	2	0.3
Basal cell skin cancer	6	0.8	Esophageal cancer	2	0.3
Endometrial cancer	4	0.5	Others	15	11.7
Prostate cancer	4	0.5			

**Figure:**
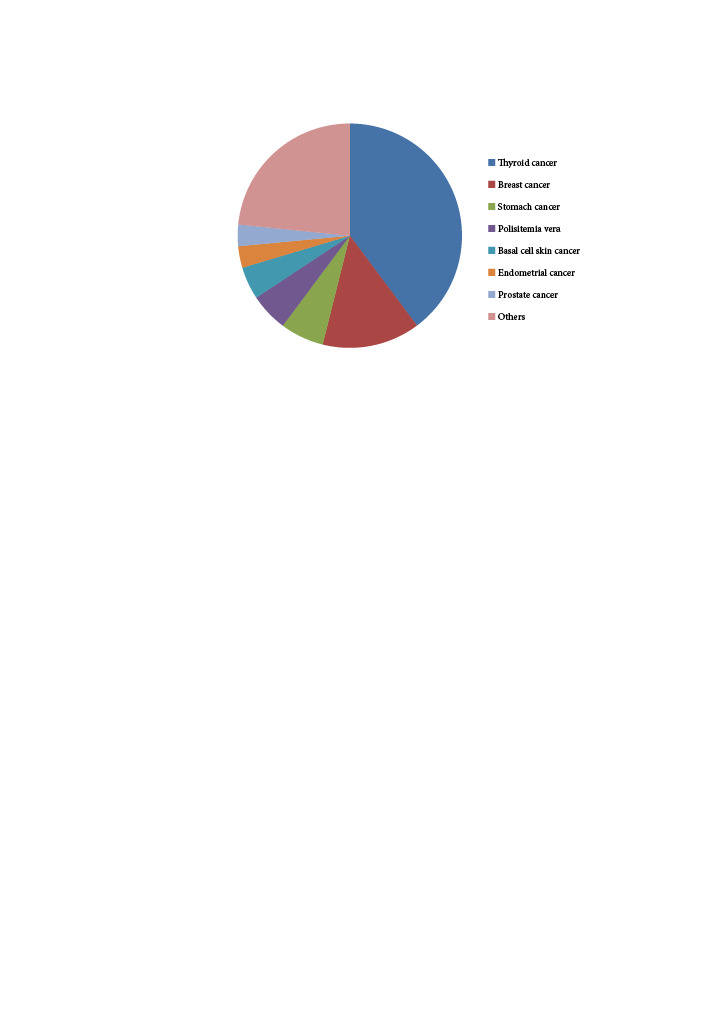
Cancer types seen in PHPT patients.

Of the 775 PHPT patients, 273 had thyroid nodules. The mean number of nodules was 1.96 ± 0.95, and 242 of them were solid and 31 of them were cystic nodules. Of the nodules, 31 were anechoic, 96 were hypoechoic, 127 were isoechoic, and 19 were hyperechoic. Mean nodule size was found as 12.44 ± 8.15 mm.

Thyroid surgery was performed in 90 patients concurrently with PHPT surgery, 7 of these 90 patients were subjected to thyroid surgery due to preoperative malignant fine-needle aspiration biopsy results, and 83 due to multinodular goiter. Postoperative final thyroidectomy pathology results were benign in 65 patients and malignant in 25 patients.

There was no significant difference between those with and without malignancy in PHPT patients in terms of age, sex, follow-up time and biochemical parameters such as Ca, P, PTH, vitamin D, creatinine and urinary Ca level. The age at the time of diagnosis of parathyroid adenoma was higher in those with malignancy than those without malignancy (p = 0.019) (Table 3).

**Table 3:** Comparison of demographic and laboratory parameters of PHPT patients with and without accompanying malignancies.

	With malignancy(n=128)	Without malignancy(n=647)	p
Age, years	63.02 ± 11.43	59.62 ± 13.30	0.067
Sex (female /male), n	99/29	526/121	0.301
Age at diagnosis, years	57.99 ± 10.86	55.14 ± 12.89	0.019
Follow up time (years)	5.09 ± 3.53	4.58 ± 3.51	0.132
Ca, mg / dL	11.29 ± 0.82	11.36 ± 0.81	0.378
Albumin-corrected Ca, mg / dL	11.13 ± 0.92	11.12 ± 0.88	0.871
P, mg / dL	2.76 ± 0.66	2.72 ± 0.63	0.516
PTH, ng / L	239.2 ± 229.53	218.3 ± 171.84	0.247
Vitamin D, ug / L	15.57 ± 10.72	14.84 ± 9,65	0.494
Creatinin, mg / dL	0.76 ± 0.27	0.77 ± 0.26	0.769
24-hour urine calcium, mg / day	322 ± 176	352 ± 180	0.171

## 4. Discussion

According to the results of our study, 128 (16.5%) of 775 PHPT patients were found to have an accompanying malignancy. The most common accompanying malignancies were determined as thyroid cancer, breast cancer and stomach cancer (51 (6.5%), 18 (2.3%) and 8 (1.0%) of patients, respectively).

Several studies have shown that PHPT patients have an increased risk of malignancy that persists after surgery. The increased risk may be associated with hypercalcemia itself. High calcium levels may increase the risk of cancer by inducing mitotic activity. There are also some experimental studies suggesting that PTH has a tumor-promoting effect and an apoptosis-inhibiting effect. However, the long-term persistence of the postoperative cancer risk has reduced the likelihood of biochemical irregularities that contribute to the risk of cancer and focused on other likely mechanisms. Vitamin D inhibits angiogenesis and invasiveness, induces apoptosis, cell cycle arrest and differentiation, thus, exhibiting an anti-tumor effect, and vitamin D receptors are present in many different cells. Defects in the vitamin D receptor alleles cause a genetic predisposition. This genetic predisposition may cause impairment in the regulation of the functions of the parathyroid glands, as well as impaired apoptosis and an increase in preneoplastic lesions [5,11].

According to the GLOBOCAN 2018 data, in both sexes combined, lung cancer is the most commonly diagnosed cancer (11.6% of the total cases), closely followed by female breast cancer (11.6%), prostate cancer (7.1%), and colorectal cancer (6.1%) [12]. In 2018, the incidence of all cancers in our country was determined as 225.1 per 100.000 persons and lung cancer (16.5%), breast cancer (10.6%), colorectal cancer (9.5%), prostate cancer (8.2%), thyroid cancer (6.2%) and stomach cancer (5.0%) constitute the first 6 types of cancer in order of frequency [12]. In our study, the rate of malignancy in PHPT patients was found to be 16.5%, and it was found to be at a higher frequency than the general population. In addition, unlike the general population, thyroid cancer was found to be the most common accompanying malignancy in PHPT patients.

A study including 9782 operated patients from Sweden showed an increased risk of cancer in both sexes, with risk persisting for more than 15 years after surgical correction of PHPT. The authors reported that breast cancer accounts for a quarter of all cancer cases in women, with an increased risk of colon, kidney, and squamous cell cancer in both sexes. An interesting result found in that study is that the risk of endocrine and pancreatic cancer is increased in the small subgroup operated before the age of 40 [5]. In our study, breast cancer was seen in 2.3% of PHPT patients and ranked second after thyroid cancer among cancers cases seen in PHPT patients.

In a study involving 3039 PHPT patients, Ghosh et al. found an increased incidence of cancer cases in patients who were conservatively managed or surgically treated, and reported that the most common types of cancer were colon, breast, lung and skin (non-melanoma) cancers. The authors also reported that surgery delays the occurrence of cancer but does not reduce it [7]. In our study, both surgically and conservatively managed patients were included, and separate evaluation has not been made for those who underwent surgical treatment and were followed up conservatively. However, in our study, unlike Ghosh et al., the most common cancers were those of thyroid, breast and stomach.

In the literature, the prevalence of thyroid carcinoma with concomitant PHPT ranges from 1% to 36 [13–20]. In their study investigating the prevalence of thyroid disease and thyroid malignancy in patients who underwent surgical treatment for PHPT, Çuhaci et al. found the thyroid malignancy prevalence as 20.8% (all patients with malignant disease had **papillary thyroid cancer**) [13]. In a similar study, Preda et al. reported the incidence of thyroid cancer as 13.6% in PHPT patients who underwent surgery [21]. In our study, the frequency of thyroid cancer in PHPT patients was found to be 6.5%, but, unlike those studies, we did not separately evaluate PHPT patients who underwent surgical treatment.

Previous studies have investigated several predisposing factors for papillary thyroid cancer in PHPT patients, such as the tumor promoting effect of PTH [22], the goitrogenic effect and increased mitotic activity induced by hypercalcemia [5,23,24] and neck irradiation [5,24,25]. A presumed role for PTH excess in triggering the onset of papillary thyroid cancer remains controversial [26]. In the study of Preda et al., although PTH levels were much higher in patients with secondary hyperparathyroidism, the similar incidence of papillary thyroid cancer in patients with secondary hyperparathyroidism and PHPT is contrary to the opinion that high PTH level may trigger papillary thyroid cancer [21]. Pickard et al. found that cancer risk in patients with secondary hyperparathyroidism was insignificant, and, in the light of this data, they suggested that PTH itself is not the cause of malignancies [27].

The limitations of our study are its small sample size, single center and retrospective design.

Our study confirms the earlier findings of a connection between PHPT and increased risk of malignancy. It is not clear whether this increased risk is due to genetic predisposition to tumor development or whether it is a physiological associative effect, intrinsic or environmental. For all these reasons, PHPT patients should be examined and monitored more carefully for all cancers, especially thyroid cancer. More comprehensive studies are needed to clarify the relationship between PHPT and cancer.

## Informed consent

Informed consent was obtained from all participants. Ethics committee approval was obtained from Necmettin Erbakan University Meram Medical Faculty Ethics Committee for the study.
